# Premature Mortality for Chronic Diseases in the EU Member States

**DOI:** 10.3390/ijerph16204021

**Published:** 2019-10-21

**Authors:** Silvia Megyesiova, Vanda Lieskovska

**Affiliations:** Faculty of Business Economics with Seat in Košice, Tajovského 13, University of Economics, Bratislava, 04130 Košice, Slovakia; vanda.lieskovska@euke.sk

**Keywords:** standardized death rates, premature mortality, chronic diseases, European Union, trend, variability, gender gap

## Abstract

Premature mortality, and especially premature mortality for chronic diseases, is a very important topic of public health, health care, or lifestyle of population. The main aim of countries is to reduce premature mortality, and therefore an analysis of the development and status of premature standardized death rates (SDR) is key for disclosure of successes or failures in this topic. A boxplot chart was used to detect extremes of SDR for both sexes. The gender ratio revealed the differences of mortality rates between men and women. Premature mortality declined steadily in the EU between 2000 and 2016. The men’s premature SDR decreased from 390 to 275.9 between 2000 and 2016, while the women’s rate declined from 180.1 to 138.2. On average, annual premature SDR dropped by 2.14% for men and 1.64% for women. Thus, the gender ratio (male/female) declined from 2.17 in 2000 to 2.0 in 2016, which is a positive change for gender gap closing. The highest proportion of premature mortality belonged to mortality for malignant neoplasms, where the rate was as high as 47% for women and 32% for men in 2016. Premature mortality for chronic disease is especially high in the “new” EU member states.

## 1. Introduction

Analysis of mortality, changes of mortality, and mortality by causes of death are especially important in the case of premature mortality [1,2]. Premature mortality is a useful indicator of population health and performance of the health care system [3]. A death is considered as premature if it occurs before the age of 65 [4,5]. Special attention is given to changes of mortality for noncommunicable diseases (NCDs). The noncommunicable diseases, or so-called chronic diseases, are diseases of long duration and are largely caused by unhealthy lifestyle or risky behaviors [4,6]. Among the main NCD risk factors are tobacco use, unhealthy diet, harmful use of alcohol, insufficient physical activity, and air pollution [6,7]. This unhealthy lifestyle results in metabolic changes, like obesity, raised blood pressure, or raised cholesterol or blood glucose levels [6,8]. Countries focus on the decline of premature mortality for NCDs, and this aim is also a part of the Sustainable Development Goals [9,10,11]. Between the targets for Goal 3, a reduction of premature mortality from NCDs by one third was set due to prevention, treatment, and promotion of wellbeing until 2030 [9]. Already in 2005, the European Union countries adopted a green paper [12], and in 2007 a white paper [13] on promoting healthy diets, physical activity, and a strategy on nutrition as a prevention of overweight, obesity, chronic diseases, and related health issues. Death from preventable diseases can be controlled and eliminated [14]. Cardiovascular diseases are the main cause of death in Romania, for example [15]. Researchers are paying attention to the analysis of factors that influence the morbidity and mortality from NCDs. Mortality analysis was oriented also to evaluation of the air pollution effect on well-being and health [16], to the definition of the relationship between medical equipment and preventable mortality [17], to the identification the changes of cardiovascular mortality through changes in medicine utilization, socioeconomic, and health system factors [18], or to the estimation of the risk of chronic diseases and death due to life satisfaction [19].

The analysis of development, trend, and status of overall premature mortality, and particularly premature mortality for chronic diseases, can help to discover successes or failures in the expected reduction of premature mortality, and can help to search for and promote useful steps in achieving a significant decline of premature mortality.

The main aim of the article is to analyze the changes of premature standardized death rates (SDR) and premature SDR for chronic diseases for both sexes in the European Union member states. Due to different mortality rates for men and women, the analysis was conducted for both sexes separately from 2000 until 2016. The analysis ended in 2016 due to the data availability in the Eurostat database [20]. Analysis was conducted for the average SDR values of the 28 member countries (EU-28), as well as for each of the EU member states separately. The analysis focuses on cumulative relative changes between 2000 and 2016, on annualized relative changes, as well as on gender disparity. The variability of premature SDR was watched in order to decide whether there exists a sign of convergence or divergence of premature SDR in the EU area. Premature mortality, and especially premature mortality for chronic diseases, is an issue for us all; everybody is responsible for their effort to live better and healthier lives.

## 2. Materials and Methods

For analytical purposes the standardized death rates were used. The SDR is a weighted average of age-specific mortality rates [21] which considers that countries with larger shares of older population also have higher death rates [4], and this approach improves comparability over time and between countries. The weighting factor is the age distribution of the European standard population [22]. Premature SDR is expressed per 100,000 inhabitants. It is calculated for the 0–64 age group as a rate of total number of deaths for people aged less than 65 in a given period to the total population aged less than 65 [21].

Premature SDR due to chronic diseases is measured by dividing the number of persons younger than 65 dying due to a chronic disease by the total population under 65. Chronic diseases include [23] malignant neoplasms (codes of the International Classification of Disease C00–C97), diabetes mellitus (E10–E14), ischemic heart diseases (I20–I25), cerebrovascular disease (I60–I69), chronic lower respiratory disease (J40–J47), and chronic liver disease (K70, K73, K74). All mortality rates come from the Statistical office of the European Union, i.e., Eurostat [20].

Analysis of development was conducted by measures that allow to monitor the development of premature mortality in the EU-28 and separately in the EU member states.

A box plot chart highlighted important distributional features of premature SDR. This diagram presents a five-number summary of the data set: minimum, 25th percentile (lower quartile), median, 75th percentile (upper quartile), and the maximum [24,25]. The box ends with lower and upper quartiles, and within the box a horizontal line indicates the median. Lines drawn from each end of the box define the maximum and/or the minimum values, but only in the case where they are within the lower and upper fence. The lower fence is defined as the 25th percentile minus 1.5 times the interquartile range (interquartile range = upper quartile minus lower quartile), while the upper fence is set as the 75th percentile plus 1.5 times the interquartile range. Any observations without these limits are considered as outliers, as extreme values, and are marked as small squares, as individual points. The box plot was used for graphical examination of the data sets and comparison of the variability in mortality rates for men and women in chosen periods.

To describe the variability of the data set, a few measures of variability were used:

- Range—the difference between the highest and the lowest values,
- Interquartile range (IQR)—the difference between the upper and the lower quartiles,
- Coefficient of variation—a relative measure of variability defined as the ratio of standard deviation to the mean. It can be expressed as a percentage and is often used as a sigma convergence measure [26].

The time series of the premature SDR data set was recorded between 2000 and 2016 on an annual basis. The mortality rates are numeric variables and therefore absolute, and relative changes can be calculated to analyze the changes of the SDR in selected years [27,28,29].

For analytical purposes, the changes of SDR were followed by cumulative change over time and by annualized change, which can be written by the following formula [30]:

(1) $$\mathrm{Cumulative}\;\mathrm{relative}\;\mathrm{change}:\;\frac{SDR_{2016}}{SDR_{2000}}-1$$

(2) $$\mathrm{Cumulative}\;\mathrm{relative}\;\mathrm{change}\;\mathrm{in}\;\%:\;\left(\frac{SDR_{2016}}{SDR_{2000}}-1\right)\cdot 100$$

(3) $$\mathrm{Annualized}\;\mathrm{relative}\;\mathrm{change}:\;{\left(\frac{SDR_{2016}}{SDR_{2000}}\right)}^{\left(1/16\right)}-1$$

(4) $$\mathrm{Annualized}\;\mathrm{relative}\;\mathrm{change}\;\mathrm{in}\;\%:\;\left({\left(\frac{SDR_{2016}}{SDR_{2000}}\right)}^{\left(1/16\right)}-1\right)\cdot 100$$

The differences between men’s and women’s death rates exist, and therefore it is important to follow to the gender gap and the gender specific disparity using a suitable statistic [30,31]. For analytical purposes, the country-specific gender disparity was monitored using the gender ratio value:

(5) $$\mathrm{Gender}\;\mathrm{ratio}:\;\frac{SDR_{M}}{SDR_{F}}$$

## 3. Results

Detailed analysis of premature SDR for all causes of death and for chronic diseases in the EU countries is presented in this section of the article.

### 3.1. Premature Standardized Death Rate for All Causes of Death

**Figure 1:**
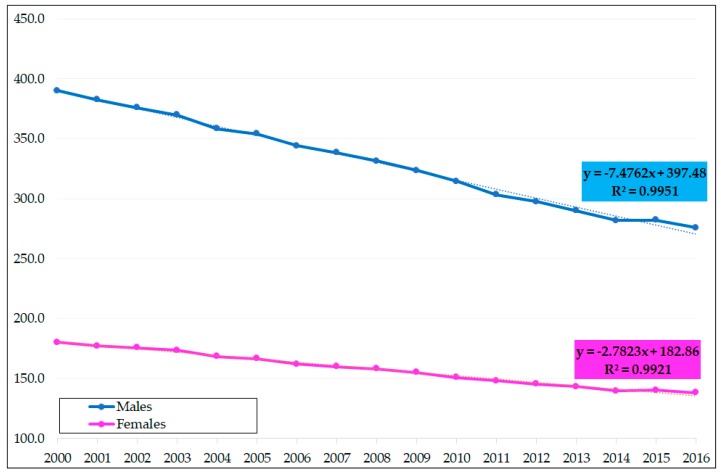
Premature standardized death rates (SDR) in 28 EU member countries (EU-28) for males and females including linear regression lines. Source: Own calculations based on Eurostat database [20].

Premature SDR in the EU declined steadily. The decrease was higher for men compared to women (see Figure 1). In EU-28, premature SDR was as high as 390 for males and 180.1 for females in 2000, meaning that the gender gap of SDR reached 209.9. The gender gap gradually decreased, with the lowest level of 137.7 in 2016. The gradual decrease of the gender gap was possible due to a rapid decline of premature SDR for males. According to the linear regression lines, premature SDR between 2000 and 2016 showed an annual average decrease of mortality rates by 7.476 for men and 2.782 for women. Due to this strong annual decrease of SDR for males compared to moderate decline for the SDR for females, the gender gap is slowly closing. In 2016, premature SDR for males ended with 275.9 compared to 138.2 for females. This progress of consecutive decline of premature SDR for both sexes should be rated very positively, and also positive is the consistent decline of the gender differences.

**Figure 2:**
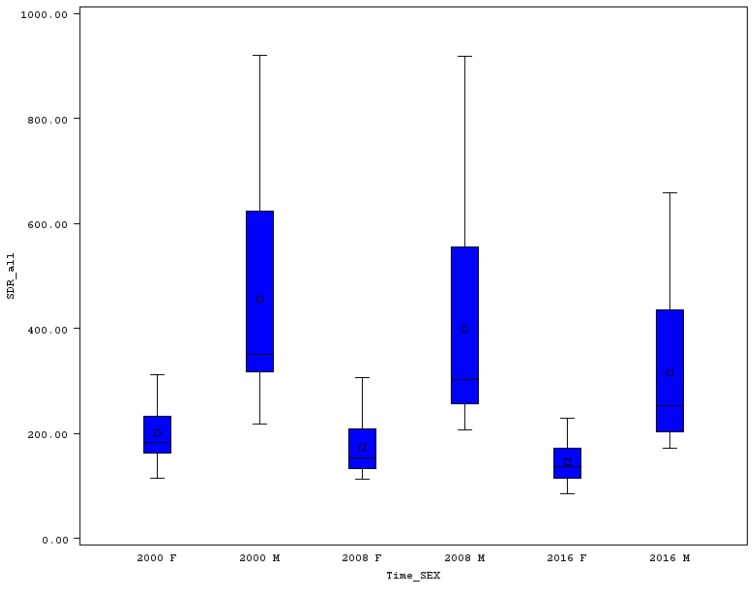
Boxplot of premature SDR in EU countries for males and females in selected years. Source: Own calculations based on Eurostat database [20].

In almost all “old” EU member states, premature SDR was lower than the EU-28 average, while in the “new” EU countries the SDR was higher (see Table 1). The variability of SDR was significantly higher for men compared to women. A very good picture of the variability is shown in the boxplot (Figure 2), where the distances of minimum and maximum, or distances between the first and third quartiles, are plotted. In 2000, the lowest SDR for males was achieved in Cyprus (218.5), while the worst in Latvia (920.5), so the range reached an extremely high level of 702 and the relative variability measured by coefficient of variation (CV) exceeded 50%. A positive picture of SDR development from 2000 until 2016 is presented in Table 1 using the cumulative and annualized relative changes of premature death rates. In Estonia, for example the overall premature SDR for men declined by 47% (from 882.1 to 467.1). In each of the EU countries, a decline for the men’s SDR was reached, which should be rated very positively. The smallest reduction in Greece was 16.4% (from a starting level of 312.1 to 261). In 2016 the best three positions with the lowest premature SDR for males belonged to Sweden (171.4), Italy (175.3), and Cyprus (178). On the other hand, the worst positions with the highest SDR were occupied by Romania (534.8), Latvia (617.4), and Lithuania (658.3).

The variability of premature SDR for females is not as high as for males. The highest death rate for females in 2000 was seen in Latvia (311.5) and the lowest in Cyprus (115.3), which resulted in a range of 196.2 and the CV was 32.4%. The highest decline of women’s premature SDR, was like in men’s SDR, achieved in Estonia (drop of 44.3%, from 304.7 to 169.8). The situation did not change for the lowest decline either, which was measured for Greece. In Greece, premature SDR for females descended from 130.4 to 118.3 (by 12.1%). The position of the EU countries according the SDR for women did not change a lot from 2000 to 2016. Cyprus in 2016 was also leading with the lowest SDR for females (85.8), followed by Spain (98.7) and Italy (99.8). The highest SDR for females belonged to the “new” member states: Latvia (221.4), Hungary (224.8), and Bulgaria (228.2). Variability changes had a good direction, as the range of premature SDR for females declined to 142.4 and the CV did not even reach the level of 30%.

The gender ratio of premature SDR was significantly higher in the former communist countries (see Table 1). In Lithuania, the gender ratio indicates that the SDR for men is about three times higher compared to women.

### 3.2. Premature Standardized Death Rate for Malignant Neoplasms

Premature SDR for malignant neoplasms (MN), when compared to other death rates for chronic diseases, is the highest one for men and for women. The lowest levels were again typical for “old” EU members, and high mortality for MN is usually seen in the “new” members states.

In Cyprus, the men’s SDR for MN was only 55.0 in 2000, while in Hungary the rate overstepped 225 (see Table 2). For EU-28 countries, the males’ premature SDR for MN declined by 27.1% between 2000 and 2016, and in ten countries the decline was higher than 30%. In the Czech Republic, premature SDR for malignant neoplasms dropped by the highest level of 45.4%. However, unfortunately, not in every EU country was such a positive development achieved. In Romania, for example, the SDR increased by 0.7%.

Cyprus was the leader, with the lowest premature SDR for MN, not only for men, but also for women, with the rate at only 53.1 in 2000 and declining by 18.7% until 2016. In just two countries was the women’s premature SDR for MN higher than 100, namely in Denmark (105.4) and Hungary (116.2) in 2000, and in only one country in 2016 (Hungary, 101.5). The best country with the highest drop in women’s premature SDR for MN was Luxembourg (39.7%). However, again, not in all countries was the change so positive. For example, in Greece the rate increased quite dramatically by 5.3%, and in Bulgaria by a moderate level of 0.2%. The average decline for the EU-28 was 10 percentage points (p.p.) lower for women than for men. The smaller relative decrease of the women’s SDR for MN is mainly caused by lower values of this SDR. In 2000, the average EU-28 SDR for men was 120.8, and it declined to 88.1 (decrease of 27.1%), while for women the rate changed from 78.3 to 65.0 (decrease of 17.0%).

**Figure 3:**
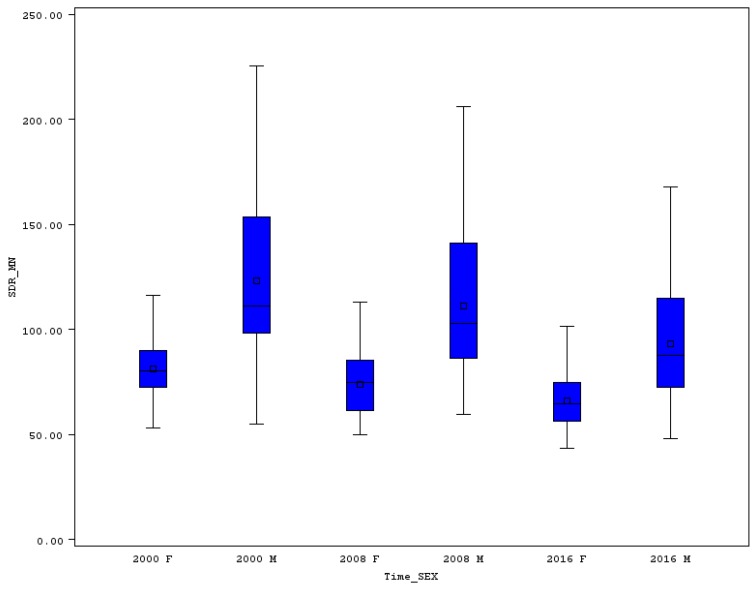
Boxplot of premature SDR for malignant neoplasms in EU countries for males and females in selected years. Source: Own calculations based on Eurostat database [20].

The variability of premature SDR for MN for men, measured by range, decreased from 170.3 in 2000 to 120.1 in 2016 (see Figure 3), and the IQR changed from 55.2 to 42.2 in the same time span. However, the relative measure of variability increased slightly from 30.9% to 33.8%. Some countries achieved very good results in the reduction of men’s SDR for MN in an analyzed time span of 17 years, and in some countries the decline was only moderate, or in the case of Romania, an increase was recorded. This is the main reason for the increased relative variability. Very similar was the change of variability in the case of women’s premature SDR for MN. We can see a small decline of the range, from 63.1 in 2000 to 58.3 in 2016, but an increase of the IQR from 17.8 to 18.6, respectively. The CV changed slightly from 18.2 to 19.0 in the same years. The variability did not change in a positive way mainly due to a strong SDR for MN decline in some of the EU countries on the one side, and a moderate decline or even increase in other EU member states. Greece and Bulgaria could not fight positively with the malignant neoplasms illnesses as premature women’s death rates in these countries increased.

The gender ratio was not as expected in a small number of countries. Usually, it is expected that the SDR for males is higher than the SDR for females. In the case of premature mortality for MN in Sweden at the beginning of the analyzed period, a different picture was the reality. The men’s SDR for MN reached 69.6 and women’s was 77.0, so the gender ratio in Sweden reached 0.9. In other countries, the gender ratio was higher than one, according the expectations. The highest ratio in 2000 was seen in Slovakia and Spain, when the men’s premature mortality was twice as high as the women’s mortality. In most of the countries the gender specific ratio declined until 2016.

### 3.3. Premature Standardized Death Rate for Ischemic Heart Diseases

**Figure 4:**
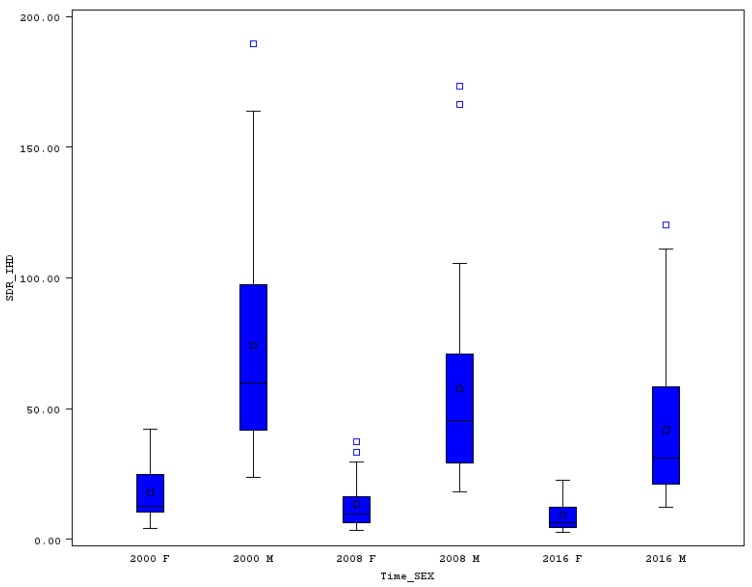
Boxplot of premature SDR for ischemic heart diseases in EU countries for males and females in selected years. Source: Own calculations based on Eurostat database [20].

The relative variability of premature mortality for ischemic heart diseases (IHD) is very high; the CV is, in most of the analyzed years, higher than 80% for both sexes. The high variability is the main reason why in the boxplot (see Figure 4), outlier values were identified for the SDR of these illnesses. In 2000 in Latvia, the men’s death rate of 189.6 was extremely high, as its value is without the whiskers of the boxplot figure. However, premature SDR for IHD was also high in other Baltic countries, such as Estonia (163.6) and Lithuania (140.3). In the same year, the rate was significantly lower in France (23.7), Portugal (31.0), or Italy (32.0). High differences between EU countries appeared also in the case of women’s SDR for IHD, where in France the level was as low as 4.2 but in Latvia it stood at 42.2. No extreme value was discovered in 2016 for females’ SDR for IHD, and one for males’ rate in Lithuania (120.4). The Baltic countries definitely have very negative results in premature SDR for IHD for both sexes. The relative changes in the case of Estonia bring at least a positive signal for a strong decline of premature men’s, and also women’s, death for IHD.

The highest relative shrinkage for men’s SDR was achieved in the Netherlands (69.8%), followed by Estonia (62.9%) and Poland (58.6%). The highest decline of women’s SDR for IHD was achieved in Estonia (75.7%), Denmark, and the Netherlands, both by 66.2%. Moderate changes were achieved paradoxically in Lithuania, where the SDR for men declined from 140.3 to 120.4 (only by 14%), which is still an extremely high death rate (see Figure 4). Low relative decline was also achieved in Greece (20.1%) and Portugal (22.0%), but we must take into account that in Portugal the death rate is not very high, so we cannot expect a further strong decline. The problem of why the relative variability of premature SDR for IHD is still so high, and why no sign of convergence is still seen, is the fact that high declines were achieved in countries with relatively low SDR (the Netherlands for example) on the one hand, and only moderate declines in countries with high death rates (Lithuania) on the other hand. A similar reason for high variability at the end of the analyzed period was discovered also for women’s SDR from IHD. The lowest declines of females’ premature mortality from IHD were measured in Greece (17.4%), Lithuania (23.7%), and France (34.7%). In the case of France, it is necessary to mention that the change still means a decline from a very low level of 4.2 to 2.8, which must be rated very positively.

The gender ratio presented in Table 3 returns a picture of huge sex differences of the SDR for IHD. In Luxembourg in 2000, the SDR for men was eight times higher than for women, but also in other EU countries the gender ratio was very high.

### 3.4. Premature Standardized Death Rate for Cerebrovascular Diseases

**Figure 5:**
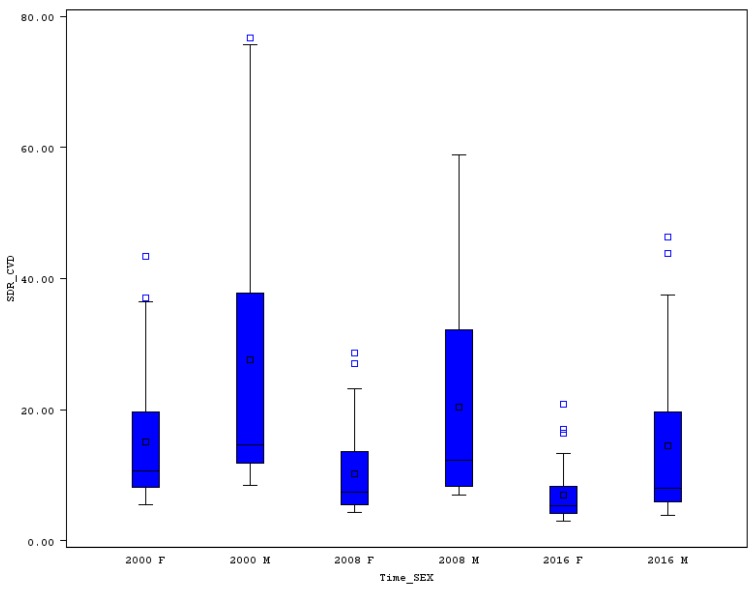
Boxplot of premature SDR for cerebrovascular diseases in EU countries for males and females in selected years. Source: Own calculations based on Eurostat database [20].

The relative variability of premature SDR for cerebrovascular disease (CVD) was the highest among other death rates for chronic diseases. The coefficient of variation for males’ SDR was higher than 100% in each year of the analyzed period. For women, the CV did not exceed 100% but was also very high compared to other death rates. In Figure 5, the boxplot chart did not identify any outlier in only one case. Extreme values were identified for females more often than for males. In 2000, the two highest extremes of premature SDR for women were seen in Romania (43.4) and Latvia (37.1). In the same year in France, the women’s premature SDR for CVD was only 5.5, in Spain 6.5, and Germany 6.6. This means that the differences among the EU countries were very high; the range stood at 37.9 in 2000. The range declined to 17.8 and the IQR dropped to 4.1 in 2016, but still the CV was at 81.2%. In 2016, the extremely high women’s values (see Figure 5 and Table 4) of premature SDR for CVD belonged to Romania (16.5), Latvia (16.9), and Bulgaria (20.8). The smallest death rates in the same year were only at about 3 in Cyprus (3.0), Sweden (3.2), and Spain (3.4). A relative decline of the women’s premature SDR for CVD between 2000 and 2016 was very strong in the case of Estonia (79.7%), Luxembourg (72%), or Ireland (70.6%), but only very moderate in Slovakia (1.7%).

The men’s premature SDR for CVD was as high as 76.7 in Bulgaria in 2000. This value was extremely high compared to the death rates of other EU countries (see Figure 5). However, the rate was also high also in Romania (75.6), Latvia (68.0), and Estonia (66.0). Again, the worst ranks of the men’s premature SDR belonged to the former communist countries (see Table 4), while the smallest death rates were achieved in Malta (8.4), France (10.2), and Sweden (10.4). The range of death rates in 2000 was 63.8, and dropped to 42.4 in 2016. Unfortunately, the CV increased in the same years from 108.8% to 116.7%. The two extreme values of premature SDR from CVD for men in 2016 belonged to Latvia (43.9) and Bulgaria (46.3). These mentioned rates are really very high when compared with the death rates of Luxembourg (3.9), Austria (5.1), and Sweden (5.4). When speaking about the decline of the men’s premature SDR for CVD between 2000 and 2016, the winner is Estonia with a decline of 74% (from 66.0 to 17.2), followed by Luxembourg (66.9%) and Slovenia (64.7%). On the other hand, during the same period only a moderate drop of death rate was achieved in Slovakia and Lithuania. In Slovakia, the SDR dropped from 28.6 to 25.1 (12.2%), and in Lithuania from 36.4 to 35.0 (3.8%).

The gender ratios were not very high. The highest gender differences appeared in the post-communist countries.

### 3.5. Premature Standardized Death Rate for Chronic Liver Disease

**Figure 6:**
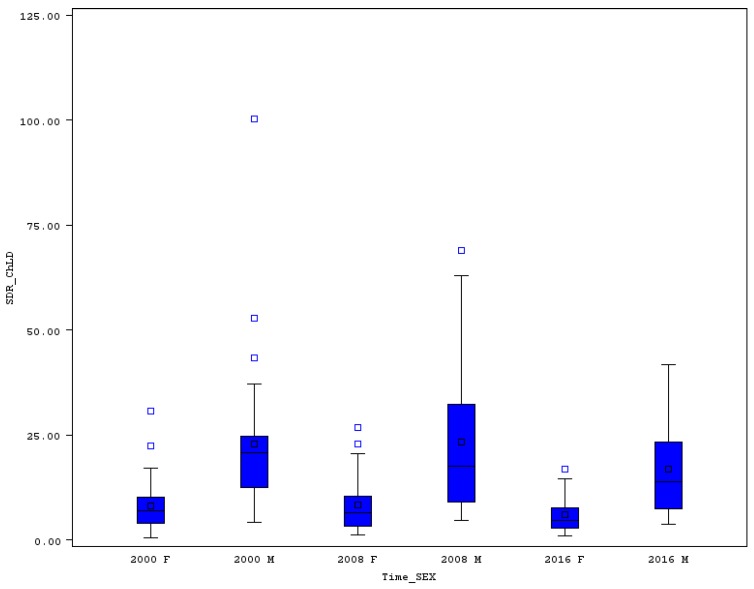
Boxplot of premature SDR for chronic liver disease in EU countries for males and females in selected years. Source: Own calculations based on Eurostat database [20].

Differences of premature SDR between the EU countries are also significantly high in the case of the death rates for chronic liver disease (ChLD). For men’s rates, three extremely high levels were identified by the boxplot chart in 2000 (see Figure 6). These extremes were seen in Slovenia (43.4), Romania (52.8), and Hungary (100.3). The range in this year stood at 96.1, while the CV was higher than 90%. In Malta, the men’s death rate for ChLD was only 4.2, in Ireland 4.7, and in Sweden 5.0. In some countries, a strong decrease between 2000 and 2016 was typical for ChLD, but unfortunately in other countries an increase was seen. In Hungary, for example, the rate decreased by 65.7%, (from 100.3 to 34.4), in Slovenia it dropped by 64.7%, and in Italy by 59.4% (from 12.4 to only 5.0). The increase of men’s premature SDR for ChLD in ten EU countries must be rated negatively, namely in Sweden, Poland, the United Kingdom, Es. altonia, Bulgaria, Greece, Finland, Latvia, Ireland, and Lithuania (see Table 5). The highest relative increase was seen in Lithuania (51.1%), Ireland (42.1), and Latvia (33.1). In 2016, no extreme was discovered by the boxplot chart, and the CV declined to 80%.

The women’s premature death rates for ChLD are lower than the men’s rates in each of the EU member states. The variability of the rates is also high, some extreme values were identified such as the case of men’s death rates for ChLD. In 2000, two extremely high levels belonged to Romania (22.3) and Hungary (30.6). This means that in Hungary, the extremely high values were reached for both sexes. The range of the death rates in this year was 30, while the CV reached quite a high level of 82%. Very low and acceptable was the premature SDR for ChLD in Malta (0.6), Greece (1.2), or Cyprus (1.8). The range declined to only 16 until 2016, but the relative measure of variability did not change a lot and was still more than 82%. Further, in 2016 one extremely high women’s death rate for ChLD was present (Romania, 16.9). Between 2000 and 2016, a strong decline of the analyzed women’s premature SDR for ChLD was achieved especially in Slovenia (81.1%), Hungary (67.5%), and Italy (64%). For Italy, the change must be rated very positively due to a high relative decline even when the death rate was already very low in 2000. The decline from 4.5 to only 1.6 in Italy was a very good result in the effort to minimize premature SDR for chronic diseases. However, not in all EU countries was such positive movement a reality. As with men’s death rates for this illness, an increase in the case of women’s premature SDR in some countries was seen. This increase concerned these countries: Slovakia (10.8%), the United Kingdom (11.4%), Poland (29.8%), Bulgaria (30.8%), Finland (37%), Lithuania (41.7%), Latvia (56.5%), Malta (70%), and Ireland (75%).

The gender ratio in some countries was higher than 5, which means that premature mortality for ChLD in these countries is more than five times higher compared to women’s rates.

### 3.6. Premature Standardized Death Rate for Diabetes Mellitus and for Chronic Lower Respiratory Diseases

**Figure 7:**
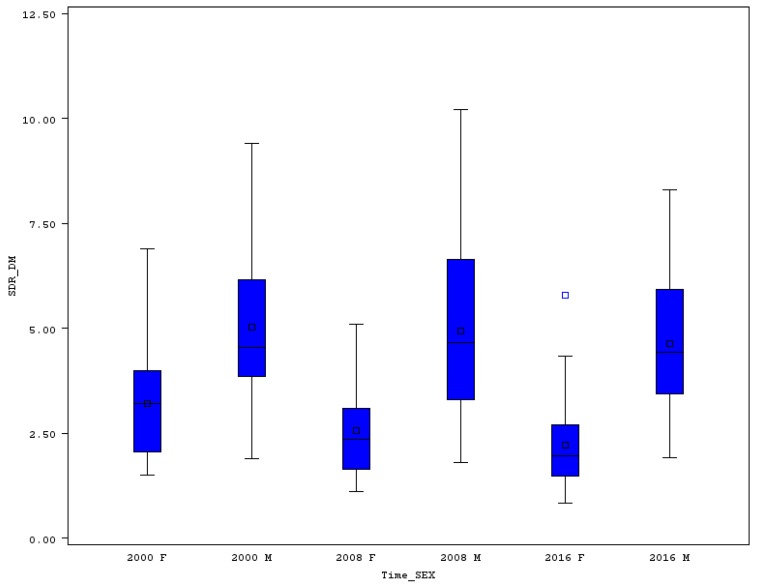
Boxplot of premature SDR for diabetes mellitus in EU countries for males and females in selected years. Source: Own calculations based on Eurostat database [20].

SDR for diabetes mellitus (DM) is not as high as the rates for other chronic diseases. Males’ premature mortality for DM ranged from 1.9 to 9.4 in 2000, and from 1.9 to 8.3 in 2016 (see Figure 7 and Table 6). The relative variability was not very high, in 2000 the CV reached 37.5% and increased to nearly 40% in 2016. The development of SDR for DM cannot be positively rated in each of the EU countries. In eleven out of 28 EU member states, the males’ SDR for DM increased between 2000 and 2016. The highest relative increase was seen in Greece (108.9%), followed by Malta (83.1%), Latvia (60.9%), Estonia (52.8%), Austria (48.1%), Luxembourg (32.7%), Czechia (28.5%), Romania (12.9%), Poland (8.7%), Croatia (7.5%), and Hungary (5.2%). On the other hand, in other countries moderate or significant decline of this SDR was achieved. The best results in reduction of premature men’s mortality for DM were achieved in Belgium (31.4%), Spain (37.2%), and the Netherlands (42%).

Females’ premature SDR for DM ranges from 1.5 to 6.9 in 2000, and from 0.8 to 5.8 in 2016. A very low mortality rate for DM in 2016 was achieved especially in Belgium and Spain (0.8), and in Luxembourg and Ireland (0.9). The SDR was higher than five only in Malta (5.8). The CV was slightly higher for women compared with men; the relative variability stood at 47.6% in 2000 and increased to 61% in 2016. The change of premature SDR for DM between 2000 and 2016 for women was not as favorable as in the case of some other chronic diseases. In six countries out of 28 EU states, the women’s SDR for DM increased, namely in Malta (60.8%), Greece (45.3%), Austria and Czechia (12.1%), Croatia (9.6%), and Latvia (0.9%). However, some countries have done very well in reducing women’s premature mortality for DM. The highest reduction was achieved in Belgium (58.3%), Lithuania (59%), and Luxembourg (74.2%).

**Figure 8:**
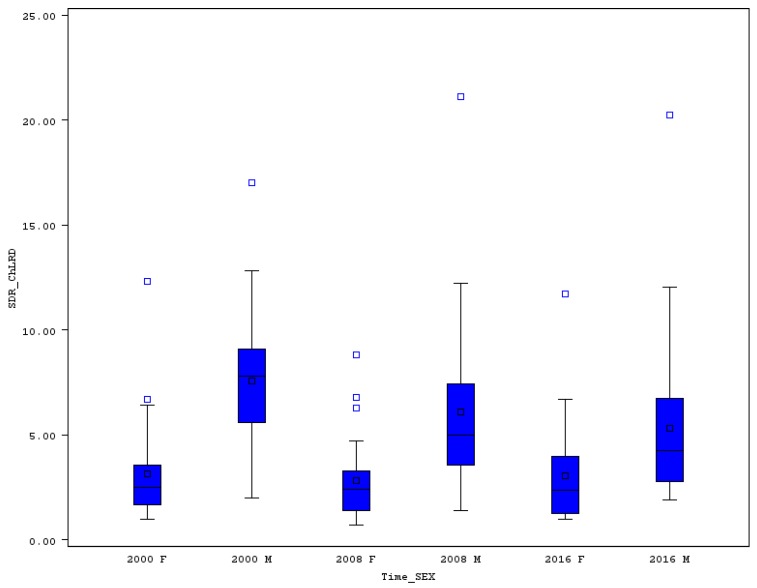
Boxplot of premature SDR for chronic lower respiratory diseases in EU countries for males and females in selected years. Source: Own calculations based on Eurostat database [20].

The men’s premature mortality for chronic lower respiratory diseases (ChLRD) in 2000 was acceptably low in Cyprus (2.0) or Greece (2.4), but higher than 12 in Lithuania (12.5), Romania (12.8), and Hungary (17.0). In 2016, premature SDR for ChLRD was only about 2 in Sweden, Cyprus, Malta, Italy, Greece, or Slovenia, but higher than 10 in Romania (12.0) and Hungary (20.3). Hungary should be negatively rated due to its extremely high levels of men’s SDR for ChLRD (see extreme values in Figure 8) and due to having the strongest relative increase of this rate (19.2%). Unfortunately, an increase of the death rates was seen also in Czechia (14.0%) and Denmark (2.5%), and a moderate increase in Germany (0.3%). The development was much more favorable in Ireland, Estonia, Luxembourg, Slovenia, and Malta where a reduction of men’s premature SDR higher than 60% was achieved.

Females’ premature SDR for ChLRD in 2000 ranged from 1.0 in Greece to extremes of 6.7 in the United Kingdom and 12.3 in Denmark (see Figure 8 and Table 7). In twelve countries, an increase of SDR for females was measured between 2000 and 2016. In Croatia, the mortality for ChLRD increased by 125.4% from a very small rate of 1.1 to 2.4. However, much more negative was the change in premature females’ mortality for Hungary, where the rate increased from 6.4 to 11.7, or by 82.8%. The next country with a high relative increase is Slovakia (65.3%), followed by Germany (47.7%), Latvia (37.1%), and Czechia (28.3%). Some countries were very successful in fighting premature SDR for ChLRD, for example in Denmark the rate decreased by 45.5%, in Malta by 62%, and in Lithuania by 64.7%.

The relative variability of premature SDR for ChLRD was in 2000 as high as 51% for men and 82% for women, and the CV increased to 71% for men and decreased to 73% for women until 2016.

## 4. Discussion

Premature mortality in some of the EU countries is still high, and the decline of this mortality is in interest of all countries, in the interest of public health and health care issues, and of course in the interest of the population all over the world. It is necessary to say that a positive movement was discovered in the decline of premature SDR for men and women and in the decline of the differences of mortality between both sexes. The variability of the overall premature mortality was higher for men compared to women. While the relative variability was about 50% in the case of men, it reached an acceptable level of about 30% for women. At the beginning of the analyzed period, the highest premature mortality for males was seen in three Baltic countries: Lithuania (804.3), Estonia (882.1), and Latvia (920.5). On the other hand, the three lowest mortality rates were achieved in the two Mediterranean island countries, Cyprus (218.5) and Malta (245.8), and Sweden (239.7). For women, the rates were much lower (see Figure 1 and Figure 2 and Table 1). The worst positions for the women’s premature SDR belonged again to the former communist countries (Hungary, Romania, and Latvia), while the best results were a reality in Cyprus, Spain, and Greece.

Is it by a coincidence that the lowest premature mortality rates were achieved in Cyprus, Spain, Malta, or Greece? Definitely not! All of these countries are well known as countries with traditional Mediterranean eating habits, and countries with the healthiest ways to eat. This Mediterranean diet concept is characterized by consumption of fresh fish foods, vegetables, fruits, and healthy fats like olive oils [32]. However, among the best countries with very low premature mortality is also Sweden. People living in Sweden practice the so called “lagom” philosophy in their everyday life for a balanced, slower, happy, and fuss-free life [33]. This combination of “lagom” with a Nordic diet, which focuses on traditional and locally sourced foods, could be the secret of low premature death rates in Sweden. In 2016, the smallest premature rates were achieved very similarly to 2016, i.e., in Sweden, Italy, and Cyprus for men, and in Cyprus, Spain, and Italy for women. The decline of premature SDR was significant in the former communist countries. In Estonia and Slovenia, the drop was even higher than 40% for both sexes. However, due to a very high starting value in 2000, premature mortality also stayed high in 2016 in Romania, Latvia, and Lithuania for men and Latvia, Hungary, and Bulgaria for women. The three Baltic countries reached the highest gender gap in 2000, while two of them (Latvia, Lithuania) and Romania had the highest gap between both sexes in 2016.

Among premature mortality for chronic diseases, the highest proportion of overall premature SDR (SDR for all causes of death) belongs to mortality from malignant neoplasms. For females, the proportion was as high as 43% in 2000 and it increased to 47% in 2016, while for men it was 31% and 32% respectively. The second highest proportion belonged to ischemic heart diseases: 13.8% in 2000 and 10.8% in 2016 for men, while for women it was 7.6% and 5.1% in the same period. The EU countries should pay close attention especially to these illnesses in order to reduce premature mortality to as low as possible.

## 5. Conclusions

The decline of premature mortality is a positive sign not only for the health care system and for public health issues, but especially for the population. The decline in some of the EU countries was strong, and in some countries the reduction was only moderate. However, unfortunately premature mortality for selected illnesses also increased between 2000 and 2016.

Countries with the lowest men’s premature SDR for MN in 2000 and 2016 are Cyprus, Sweden, and Finland. The best positions for women’s rates belong to Cyprus, Greece, and Spain in 2000 and Cyprus, Luxembourg, and Finland in 2016. This confirms the success of Mediterranean countries and Nordic countries in fighting premature mortality from MN due a healthy Mediterranean and/or Nordic diet, and the peaceful, balanced, fuss-free lifestyle of the Nordic population. The East European countries, Czechia, Slovakia, and Hungary, resulted very badly in 2000 due to their high premature mortality rates from MN for males and Estonia, Denmark, and Hungary for females. In 2016, the worst positions belonged to Romania and Hungary for both sexes. The relative variability of premature mortality from MN was not very high, about 20% for women and 30% for men.

Extreme values of premature SDR for IHD, CVD, ChLD, and ChLRD were identified both for men and women in some years (see the boxplots). This is the main reason why the relative variability of these rates is high. The CV for premature SDR from IHD was in some years even higher than 100%. In 2000, the highest premature men’s mortality rates for IHD were seen in three Baltic countries, while the lowest were in France, Portugal, and Italy. The situation did not change a lot until 2016, when the position of the highest rates belonged to Hungary, Latvia, and Lithuania. Two of the Baltic countries (Estonia, Latvia) and Romania performed very badly in 2000, with high females’ premature mortality for IHD compared with lowest rates in France, Luxembourg, and Spain. Again, France, Luxembourg and, instead of Spain, Italy, were the leaders with the lowest women’s premature SDR for IHD in 2016.

Not only was premature SDR from IHD characterized by a very high relative variability, the CV was even higher in the case of premature mortality for CVD. No big surprise was that the three worst premature SDRs for CVD in 2000 belonged to Latvia, Romania, and Bulgaria for both sexes, while the best results were achieved in Malta, France, and Sweden for men and France, Spain, and Germany for women. Here again, we can see the lowest values of mortality in the Mediterranean countries of the EU and Sweden or Luxembourg, and the worst valued in the “newest” EU member states. Premature mortality for ChLD confirms the rule of extremely high mortality in the former communist countries. The boxplot figure detected in 2000 extremely high SDR for men in Slovenia, Romania, and Hungary, and for women in Romania and Hungary. How healthy the Mediterranean diet and lifestyle is can be proven by the lowest premature women’s mortality for ChLD in Malta, Greece, and Cyprus. In exactly these three countries the premature death rate was pushed to only about 1 in 2016. The variability of premature SDR for DM was within acceptable limits, and only in 2016 one extreme value for the women’s rate was detected. Premature mortality for DM could be pushed down by a healthier diet of the population.

Romania and Hungary ranked worst both at the beginning and at the end of the analyzed period in term of the highest men’s premature SDR for ChLRD. In Hungary, premature mortality for these illnesses increased from 17.0 in 2000 to 20.3 in 2016. However, the men’s premature SDR for ChLRD increased not only in Hungary, it also went up in Czechia, Denmark, and moderately in Germany. The main aim of premature SDR reduction totally failed in the case of women’s premature mortality for ChLRD. However, in 12 out of 28 countries an increase of the mortality was attained. It is necessary to mention that the EU countries achieved very good results in the reduction of premature mortality for both sexes, especially for malignant neoplasms, ischemic heart diseases, cerebrovascular diseases, and moderate decline of chronic liver disease. Premature SDR for diabetes mellitus and for chronic lower respiratory diseases changed very slowly, and the progress for these mortalities was not as favorable as for the earlier mentioned illnesses.

The EU countries should continue their efforts for the reduction of premature mortality for chronic diseases. Furthermore, the population living in Europe should keep in mind that they are responsible for their health through healthy diet, through healthy eating habits, through sufficient physical activity, and through reduction of risk factors such as tobacco use or harmful use of alcohol. Only the combination of a well-functioning healthcare system in the EU countries and the effort of the population to reduce their risky behaviors can bring a further reduction of premature mortality for noncommunicable diseases in the EU states.

## Figures and Tables

**Table 1:** Premature standardized death rates (SDR) in the EU countries in 2000 and 2016 (all causes of death) ^1^.

EU	SDR-Males in		Relative Change in %		EU	SDR-Females in		Relative Change in %		EU	Gender Disparity Ratio		Cumulative	EU	M	EU	F
States	2000	2016	Cumulative	Annualized	States	2000	2016	Cumulative	Annualized	States	2000	2016	Change, %	States	2016	States	2016
Cyprus (CY)	218.5	178.0	−18.6	−1.27	CY	115.3	85.8	−25.6	−1.83	MT	1.52	1.67	10.4	Austria (AT)	217.9	Austria (AT)	118.4
Sweden (SE)	239.7	171.4	−28.5	−2.07	ES	129.0	98.7	−23.5	−1.66	DK	1.53	1.57	2.4	Belgium (BE)	236.0	Belgium (BE)	137.8
Malta (MT)	245.8	193.0	−21.5	−1.50	EL	130.4	118.3	−9.3	−0.61	SE	1.57	1.55	−1.1	Bulgaria (BG)	527.8	Bulgaria (BG)	228.2
Italy (IT)	275.8	175.3	−36.4	−2.79	IT	136.8	99.8	−27.1	−1.95	NL	1.57	1.32	−15.8	Croatia (HR)	358.5	Croatia (HR)	146.7
Netherlands (NL)	283.4	182.4	−35.7	−2.72	SE	152.9	110.5	−27.7	−2.01	UK	1.63	1.53	−6.0	Cyprus (CY)	178.0	Cyprus (CY)	85.8
United Kingdom (UK)	303.0	226.7	−25.2	−1.80	FR	155.1	126.6	−18.4	−1.26	IE	1.72	1.62	−5.8	Czechia (CZ)	310.2	Czechia (CZ)	145.1
Greece (EL)	312.1	261.0	−16.4	−1.11	MT	162.2	115.3	−28.9	−2.11	LU	1.87	1.87	0.2	Denmark (DK)	229.7	Denmark (DK)	146.5
Luxembourg (LU)	322.1	199.8	−38.0	−2.94	AT	162.5	118.4	−27.1	−1.96	CY	1.90	2.08	9.5	Estonia (EE)	467.1	Estonia (EE)	169.8
Spain (ES)	325.0	207.4	−36.2	−2.77	FI	162.9	114.9	−29.5	−2.16	BE	1.94	1.71	−11.5	EU-28	275.9	EU-28	138.2
Ireland (IE)	329.2	194.4	−40.9	−3.24	DE	166.2	137.7	−17.2	−1.17	IT	2.02	1.76	−12.8	Finland (FI)	251.4	Finland (FI)	114.9
Portugal (PT)	334.9	229.7	−31.4	−2.33	LU	172.6	106.9	−38.1	−2.95	DE	2.03	1.86	−8.7	France (FR)	263.6	France (FR)	126.6
Austria (AT)	335.8	217.9	−35.1	−2.67	PT	174.2	115.3	−33.8	−2.55	AT	2.07	1.84	−11.0	Germany (DE)	255.9	Germany (DE)	137.7
Germany (DE)	338.2	255.9	−24.3	−1.73	BE	174.8	137.8	−21.2	−1.48	EU-28	2.17	2.00	−7.8	Greece (EL)	261.0	Greece (EL)	118.3
Portugal (PT)	338.4	236.0	−30.3	−2.23	EU-28	180.1	138.2	−23.3	−1.64	RO	2.23	2.46	10.4	Hungary (HU)	504.2	Hungary (HU)	224.8
France (FR)	361.9	263.6	−27.2	−1.96	NL	180.3	137.7	−23.6	−1.67	BG	2.27	2.31	1.7	Ireland (IE)	194.4	Ireland (IE)	120.2
Finland (FI)	374.6	251.4	−32.9	−2.46	UK	186.2	148.2	−20.4	−1.42	FI	2.30	2.19	−4.9	Italy (IT)	175.3	Italy (IT)	99.8
EU-28	390.0	275.9	−29.3	−2.14	IE	191.8	120.2	−37.3	−2.88	PT	2.32	2.41	4.0	Latvia (LV)	617.4	Latvia (LV)	221.4
Portugal (PT)	403.4	277.7	−31.2	−2.31	SI	196.8	112.1	−43.0	−3.46	FR	2.33	2.08	−10.8	Lithuania (LT)	658.3	Lithuania (LT)	209.9
Slovenia (SI)	461.0	249.4	−45.9	−3.77	HR	199.9	146.7	−26.6	−1.91	SI	2.34	2.22	−5.0	Luxembourg (LU)	199.8	Luxembourg (LU)	106.9
Czechia (CZ)	490.3	310.2	−36.7	−2.82	CZ	208.3	145.1	−30.3	−2.23	CZ	2.35	2.14	−9.2	Malta (MT)	193.0	Malta (MT)	115.3
Croatia (HR)	513.8	358.5	−30.2	−2.22	DK	218.8	146.5	−33.0	−2.47	EL	2.39	2.21	−7.8	Netherlands (NL)	182.4	Netherlands (NL)	137.7
Poland (PL)	617.4	455.1	−26.3	−1.89	SK	229.3	173.9	−24.2	−1.71	HU	2.51	2.24	−10.5	Poland (PL)	455.1	Poland (PL)	176.7
Slovakia (SK)	630.4	416.1	−34.0	−2.56	PL	237.4	176.7	−25.6	−1.83	ES	2.52	2.10	−16.6	Portugal (PT)	277.7	Portugal (PT)	115.3
Bulgaria (BG)	644.8	527.8	−18.1	−1.24	LT	271.0	209.9	−22.5	−1.58	HR	2.57	2.44	−4.9	Romania (RO)	534.8	Romania (RO)	217.4
Romania (RO)	691.0	534.8	−22.6	−1.59	BG	283.6	228.2	−19.5	−1.35	PL	2.60	2.58	−1.0	Slovakia (SK)	416.1	Slovakia (SK)	173.9
Hungary (HU)	776.6	504.2	−35.1	−2.66	EE	304.7	169.8	−44.3	−3.59	SK	2.75	2.39	−13.0	Slovenia (SI)	249.4	Slovenia (SI)	112.1
Lithuania (LT)	804.3	658.3	−18.1	−1.24	HU	309.7	224.8	−27.4	−1.98	EE	2.89	2.75	−5.0	Spain (ES)	207.4	Spain (ES)	98.7
Estonia (EE)	882.1	467.1	−47.0	−3.90	RO	310.0	217.4	−29.9	−2.19	LV	2.96	2.79	−5.6	Sweden (SE)	171.4	Sweden (SE)	110.5
Latvia (LV)	920.5	617.4	−32.9	−2.47	LV	311.5	221.4	−28.9	−2.11	LT	2.97	3.14	5.7	United Kingdom (UK)	226.7	United Kingdom (UK)	148.2
Coefficient of variation (CV) %	52.0	51.9			CV, %	32.4	29.9										

Source: Own calculations based on Eurostat database [20]. The SDR and the gender disparity ratio ordered from min to max values in 2000. Outlier values marked by asterisks. ^1^ Gender disparity ratio: (SDR_Male_)/(SDR_Female_).

**Table 2:** Premature SDR for malignant neoplasms in the EU countries in 2000 and 2016 ^1^.

EU	SDR-Males in		Relative Change in %		EU	SDR-Females in		Relative Change in %		EU	Gender Disp. Ratio		Cumulative
States	2000	2016	Cumulative	Annualized	States	2000	2016	Cumulative	Annualized	States	2000	2016	Change, %
Cyprus (CY)	55.0	52.5	−4.7	−0.30	CY	53.1	43.2	−18.7	−1.28	SE	0.90	0.89	−1.2
Sweden (SE)	69.6	47.9	−31.1	−2.30	EL	57.1	60.1	5.3	0.32	DK	1.02	1.00	−1.7
Finland (FI)	74.4	58.1	−21.9	−1.53	ES	59.3	54.0	−8.9	−0.58	CY	1.04	1.21	17.2
Malta (MT)	89.6	72.9	−18.6	−1.28	FI	64.1	50.3	−21.6	−1.51	IE	1.05	0.98	−6.4
United Kingdom (UK)	92.0	69.5	−24.5	−1.74	PT	66.1	56.0	−15.3	−1.03	UK	1.07	1.06	−1.1
Luxembourg (LU)	97.0	80.8	−16.7	−1.14	IT	69.1	55.9	−19.1	−1.32	NL	1.13	0.98	−13.1
Ireland (IE)	97.5	63.6	−34.7	−2.63	FR	69.1	61.6	−10.9	−0.72	MT	1.16	1.29	11.1
Netherlands (NL)	99.4	73.2	−26.4	−1.89	AT	75.5	58.8	−22.1	−1.55	FI	1.16	1.16	−0.4
Greece (EL)	100.3	89.5	−10.8	−0.71	DE	76.0	63.8	−16.1	−1.09	LU	1.22	1.68	38.0
Austria (AT)	101.6	73.1	−28.0	−2.04	SE	77.0	53.7	−30.3	−2.23	AT	1.35	1.24	−7.6
Italy (IT)	104.9	69.6	−33.6	−2.53	MT	77.2	56.6	−26.7	−1.92	DE	1.41	1.25	−11.4
Germany (DE)	106.9	79.5	−25.7	−1.84	BE	77.2	60.6	−21.5	−1.50	BE	1.48	1.26	−15.0
Portugal (PT)	107.2	106.5	−0.6	−0.04	EU-28	78.3	65.0	−17.0	−1.16	IT	1.52	1.25	−17.9
Denmark (DK)	107.7	72.4	−32.8	−2.45	BG	78.7	78.9	0.2	0.01	EU-28	1.54	1.36	−12.2
Belgium (BE)	114.5	76.5	−33.2	−2.49	LU	79.5	48.0	−39.7	−3.11	EE	1.57	1.44	−8.7
EU-28	120.8	88.1	−27.1	−1.96	SI	81.4	64.6	−20.7	−1.44	BG	1.57	1.49	−5.5
Spain (ES)	120.9	86.0	−28.8	−2.10	HR	81.6	74.2	−9.0	−0.59	SI	1.60	1.42	−10.8
Bulgaria (BG)	123.9	117.4	−5.3	−0.34	LV	83.1	75.4	−9.3	−0.61	PT	1.62	1.90	17.3
Slovenia (SI)	129.9	91.9	−29.2	−2.14	UK	85.7	65.5	−23.6	−1.67	RO	1.69	1.80	6.4
France (FR)	136.6	94.3	−31.0	−2.29	NL	88.1	74.6	−15.3	−1.03	PL	1.70	1.43	−16.1
Romania (RO)	151.5	152.5	0.7	0.04	SK	88.6	75.3	−15.1	−1.01	CZ	1.72	1.38	−20.1
Estonia (EE)	152.1	102.9	−32.4	−2.41	RO	89.5	84.7	−5.4	−0.34	EL	1.76	1.49	−15.2
Latvia (LV)	155.1	125.3	−19.2	−1.32	LT	90.7	76.7	−15.4	−1.04	LT	1.81	1.81	0.1
Croatia (HR)	161.4	130.6	−19.1	−1.32	IE	93.1	64.9	−30.3	−2.23	LV	1.87	1.66	−10.9
Poland (PL)	164.0	112.4	−31.5	−2.34	CZ	95.4	65.2	−31.7	−2.35	HU	1.94	1.65	−14.7
Lithuania (LT)	164.2	139.1	−15.3	−1.03	PL	96.2	78.6	−18.3	−1.26	FR	1.98	1.53	−22.6
Czechia (CZ)	164.5	89.8	−45.4	−3.71	EE	96.8	71.7	−25.9	−1.86	HR	1.98	1.76	−11.1
Slovakia (SK)	178.7	118.0	−34.0	−2.56	DK	105.4	72.1	−31.6	−2.35	SK	2.02	1.57	−22.2
Hungary (HU)	225.3	168.0	−25.4	−1.82	HU	116.2	101.5	−12.6	−0.84	ES	2.04	1.59	−21.9
Coefficient of variation (CV) %	30.9	33.8			CV, %	18.2	19.0						

Source: Own calculations based on Eurostat database [20]. The SDR and the gender disparity ratio ordered from min to max values in 2000. Outlier values marked by asterisks. ^1^ Gender disparity ratio: (SDR_Male_)/(SDR_Female_).

**Table 3:** Premature SDR for ischemic heart diseases in the EU countries in 2000 and 2016 ^1^.

EU	SDR-Males in		Relative Change in %		EU	SDR-Females in		Relative Change in %		EU	Gender Disp. Ratio		Cumulative
States	2000	2016	Cumulative	Annualized	States	2000	2016	Cumulative	Annualized	States	2000	2016	Change, %
France (FR)	23.7	14.4	−39.2	−3.06	FR	4.2	2.8	−34.7	−2.63	MT	2.48	2.27	−8.2
Portugal (PT)	31.0	24.2	−22.0	−1.54	LU	5.2	3.1	−39.6	−3.10	RO	2.97	3.72	24.9
Italy (IT)	32.0	16.8	−47.5	−3.95	ES	6.1	3.6	−41.8	−3.33	DK	3.32	4.23	27.5
Spain (ES)	36.0	19.5	−45.9	−3.77	IT	6.6	3.5	−47.0	−3.89	SK	3.40	3.89	14.4
Denmark (DK)	38.5	16.6	−56.9	−5.13	PT	8.4	4.4	−47.3	−3.92	HU	3.57	3.70	3.6
Belgium (BE)	38.8	16.6	−57.2	−5.17	SI	9.7	5.3	−45.5	−3.72	BG	3.63	4.31	18.8
Netherlands (NL)	40.8	12.3	−69.8	−7.20	BE	10.1	4.7	−53.1	−4.62	PT	3.69	5.46	47.9
Luxembourg (LU)	42.9	18.2	−57.5	−5.21	CY	10.8	5.9	−45.7	−3.74	SE	3.70	3.64	−1.5
Sweden (SE)	45.1	22.7	−49.8	−4.21	NL	11.0	3.7	−66.2	−6.55	NL	3.71	3.32	−10.6
Germany (DE)	47.7	27.2	−43.0	−3.45	EL	11.3	9.3	−17.4	−1.19	UK	3.77	3.77	0.0
Slovenia (SI)	49.8	27.5	−44.8	−3.65	DE	11.5	6.4	−44.2	−3.58	BE	3.85	3.51	−8.9
Malta (MT)	53.5	30.1	−43.7	−3.52	DK	11.6	3.9	−66.2	−6.56	EU-28	3.96	4.28	8.0
EU-28	53.8	29.9	−44.4	−3.61	SE	12.2	6.2	−49.0	−4.12	CZ	4.00	4.61	15.3
Austria (AT)	57.1	27.2	−52.4	−4.54	AT	12.3	6.0	−51.1	−4.37	HR	4.13	5.16	24.9
Cyprus (CY)	58.8	38.2	−35.1	−2.66	FI	13.0	5.2	−60.4	−5.62	DE	4.15	4.24	2.1
Greece (EL)	60.7	48.5	−20.1	−1.39	EU-28	13.6	7.0	−48.5	−4.07	PL	4.40	4.57	3.9
United Kingdom (UK)	66.0	32.1	−51.3	−4.40	IE	14.9	6.4	−56.8	−5.12	EE	4.46	6.80	52.5
Ireland (IE)	72.2	30.2	−58.2	−5.30	UK	17.5	8.5	−51.3	−4.40	LV	4.49	4.94	10.0
Croatia (HR)	77.3	56.4	−27.1	−1.96	HR	18.7	10.9	−41.7	−3.31	AT	4.64	4.51	−2.8
Finland (FI)	78.6	34.4	−56.2	−5.04	PL	21.2	8.5	−60.1	−5.59	IE	4.85	4.70	−3.1
Czechia (CZ)	88.4	44.4	−49.8	−4.22	MT	21.6	13.3	−38.7	−3.01	IT	4.85	4.80	−1.0
Poland (PL)	93.3	38.6	−58.6	−5.36	CZ	22.1	9.6	−56.5	−5.07	LT	5.05	5.67	12.4
Bulgaria (BG)	101.3	62.1	−38.7	−3.01	LT	27.8	21.2	−23.7	−1.67	SI	5.13	5.20	1.2
Slovakia (SK)	111.9	64.2	−42.6	−3.41	BG	27.9	14.4	−48.4	−4.05	EL	5.37	5.20	−3.2
Romania (RO)	115.1	73.4	−36.2	−2.77	SK	32.9	16.5	−49.8	−4.22	CY	5.44	6.49	19.5
Hungary (HU)	125.7	82.0	−34.8	−2.64	HU	35.2	22.2	−37.0	−2.85	FR	5.61	5.22	−7.0
Lithuania (LT)	140.3	120.4 *	−14.2	−0.95	EE	36.7	8.9	−75.7	−8.46	ES	5.90	5.49	−7.0
Estonia (EE)	163.6	60.6	−62.9	−6.02	RO	38.7	19.8	−48.9	−4.11	FI	6.05	6.68	10.4
Latvia (LV)	189.6 *	111.2	−41.3	−3.28	LV	42.2	22.5	−46.7	−3.85	LU	8.25	5.81	−29.6
Coefficient of variation (CV) %	77.3	92.7			CV, %	80.0	86.6						

Source: Own calculations based on Eurostat database [20]. The SDR and the gender disparity ratio ordered from min to max values in 2000. Outlier values marked by asterisks (*). ^1^ Gender disparity ratio: (SDR_Male_)/(SDR_Female_).

**Table 4:** Premature SDR for cerebrovascular diseases in the EU countries in 2000 and 2016 ^1^.

EU	SDR-Males in		Relative Change in %		EU	SDR-Females in		Relative Change in %		EU	Gender Disp. Ratio		Cumulative
States	2000	2016	Cumulative	Annualized	States	2000	2016	Cumulative	Annualized	States	2000	2016	Change, %
Malta (MT)	8.4	5.4	−35.5	−2.70	FR	5.5	3.5	−36.2	−2.77	LU	0.79	0.93	18.2
France (FR)	10.2	6.2	−39.7	−3.11	ES	6.5	3.4	−48.2	−4.02	MT	1.00	0.92	−8.0
Sweden (SE)	10.4	5.4	−48.6	−4.07	DE	6.6	4.2	−35.8	−2.73	IE	1.03	1.63	59.2
Netherlands (NL)	10.9	5.5	−50.0	−4.24	IT	6.6	3.5	−47.3	−3.92	NL	1.25	1.18	−5.4
Italy (IT)	11.0	5.5	−49.8	−4.22	SE	7.2	3.2	−55.8	−4.98	UK	1.28	1.33	3.7
Belgium (BE)	11.4	6.4	−43.7	−3.53	CY	7.5	3.0	−59.4	−5.47	DK	1.38	1.26	−8.8
Germany (DE)	11.9	6.7	−43.5	−3.51	AT	8.2	4.1	−50.5	−4.30	BE	1.38	1.34	−3.1
Ireland (IE)	11.9	5.6	−53.2	−4.63	BE	8.2	4.8	−41.9	−3.34	FI	1.39	1.71	22.8
Luxembourg (LU)	11.9	3.9	−66.9	−6.68	MT	8.4	5.9	−29.9	−2.19	SE	1.44	1.68	16.5
Spain (ES)	12.2	6.7	−45.1	−3.68	NL	8.7	4.6	−47.1	−3.90	AT	1.55	1.26	−18.9
United Kingdom (UK)	12.4	7.2	−41.9	−3.33	DK	9.7	5.6	−42.1	−3.35	IT	1.67	1.59	−4.8
Austria (AT)	12.7	5.1	−59.8	−5.54	UK	9.7	5.4	−43.9	−3.55	EU-28	1.70	1.87	10.1
Cyprus (CY)	13.0	6.6	−49.4	−4.16	EL	9.9	6.3	−36.1	−2.76	CY	1.73	2.16	24.6
Denmark (DK)	13.4	7.1	−47.2	−3.91	SK	10.1	9.9	−1.7	−0.11	RO	1.74	2.27	30.3
Finland (FI)	15.9	8.8	−44.8	−3.65	SI	11.1	4.8	−56.8	−5.12	DE	1.80	1.58	−12.1
EU-28	19.8	10.5	−46.8	−3.86	FI	11.4	5.1	−55.1	−4.88	LV	1.83	2.60	41.7
Greece (EL)	20.2	12.2	−39.7	−3.11	IE	11.6	3.4	−70.6	−7.37	FR	1.85	1.75	−5.4
Slovenia (SI)	26.6	9.4	−64.7	−6.30	EU-28	11.6	5.6	−51.6	−4.44	LT	1.88	2.64	40.5
Czechia (CZ)	27.6	11.6	−57.9	−5.26	CZ	12.9	4.8	−62.6	−5.96	ES	1.88	1.99	5.9
Slovakia (SK)	28.6	25.1	−12.2	−0.81	PT	14.5	5.6	−61.3	−5.76	PL	1.96	2.43	23.7
Portugal (PT)	29.8	12.0	−59.8	−5.54	LU	15.1	4.2	−72.0	−7.65	HR	2.03	2.31	13.6
Lithuania (LT)	36.4	35.0	−3.8	−0.24	LT	19.4	13.3	−31.5	−2.34	EL	2.04	1.92	−5.7
Poland (PL)	39.1	19.5	−50.1	−4.25	PL	19.9	8.0	−59.6	−5.51	PT	2.06	2.13	3.8
Croatia (HR)	42.7	19.7	−54.0	−4.74	HR	21.0	8.5	−59.5	−5.49	BG	2.10	2.23	5.9
Hungary (HU)	57.4	25.4	−55.7	−4.97	HU	25.7	10.8	−57.9	−5.27	CZ	2.14	2.41	12.4
Estonia (EE)	66.0	17.2	−74.0	−8.07	EE	27.7	5.6	−79.7	−9.50	HU	2.23	2.35	5.2
Latvia (LV)	68.0	43.9 *	−35.5	−2.70	BG	36.5	20.8 *	−43.0	−3.45	EE	2.38	3.06	28.5
Romania (RO)	75.6	37.4	−50.5	−4.30	LV	37.1 *	16.9 *	−54.3	−4.80	SI	2.40	1.96	−18.2
Bulgaria (BG)	76.7 *	46.3 *	−39.6	−3.11	RO	43.4 *	16.5 *	−62.1	−5.87	SK	2.83	2.53	−10.7
Coefficient of variation (CV) %	108.8	116.7			CV, %	87.0	81.2						

Source: Own calculations based on Eurostat database [20]. The SDR and the gender disparity ratio ordered from min to max values in 2000. Outlier values marked by asterisks (*). ^1^ Gender disparity ratio: (SDR_Male_)/(SDR_Female_).

**Table 5:** Premature SDR for chronic liver disease in the EU countries in 2000 and 2016 ^1^.

EU	SDR-Males in		Relative Change in %		EU	SDR-Females in		Relative Change in %		EU	Gender Disp. Ratio		Cumulative
States	2000	2016	Cumulative	Annualized	States	2000	2016	Cumulative	Annualized	States	2000	2016	Change, %
Malta (MT)	4.2	3.8	−9.0	−0.59	MT	0.6	1.0	70.0	3.37	AT	3.04	2.93	−3.6
Ireland (IE)	4.7	6.7	42.1	2.22	EL	1.2	1.0	−16.7	−1.13	BE	2.22	2.12	−4.4
Sweden (SE)	5.0	5.1	1.6	0.10	CY	1.8	0.9	−48.9	−4.11	BG	5.06	4.73	−6.6
Greece (EL)	5.1	6.3	23.7	1.34	IE	2.0	3.5	75.0	3.56	CY	3.17	4.18	32.2
Netherlands (NL)	5.4	3.8	−29.3	−2.14	NL	2.7	1.9	−30.4	−2.24	CZ	2.94	2.36	−19.6
Cyprus (CY)	5.7	3.9	−32.5	−2.42	SE	2.8	2.6	−7.9	−0.51	DE	2.49	2.51	0.8
Italy (IT)	12.4	5.0	−59.4	−5.48	ES	3.5	1.6	−55.1	−4.89	DK	2.63	2.20	−16.4
United Kingdom (UK)	12.4	13.4	7.7	0.46	IT	4.5	1.6	−64.0	−6.19	EE	2.42	2.90	19.8
Belgium (BE)	12.8	9.7	−23.7	−1.68	BG	4.8	6.3	30.8	1.69	EL	4.25	6.31	48.5
Spain (ES)	13.0	8.1	−38.1	−2.95	FI	5.6	7.7	37.0	1.99	ES	3.71	5.13	38.0
Finland (FI)	17.2	22.3	29.7	1.64	BE	5.8	4.6	−20.2	−1.40	EU-28	2.69	2.71	0.5
France (FR)	17.4	10.4	−40.2	−3.16	PL	6.0	7.8	29.8	1.65	FI	3.07	2.91	−5.3
Latvia (LV)	18.3	24.4	33.1	1.80	UK	6.3	7.0	11.4	0.68	FR	2.48	3.27	32.2
Denmark (DK)	20.5	11.2	−45.6	−3.73	PT	6.8	3.0	−55.3	−4.91	HR	3.60	4.71	30.8
EU-28	21.0	13.9	−33.9	−2.56	FR	7.0	3.2	−54.8	−4.84	HU	3.28	3.46	5.6
Poland (PL)	21.1	22.1	4.7	0.29	LV	7.7	12.1	56.5	2.84	IE	2.35	1.91	−18.8
Portugal (PT)	21.1	12.5	−40.8	−3.22	EU-28	7.8	5.1	−34.2	−2.59	IT	2.76	3.10	12.7
Luxembourg (LU)	21.9	13.9	−36.7	−2.81	DK	7.8	5.1	−34.9	−2.64	LT	2.35	2.51	6.7
Germany (DE)	22.4	13.9	−38.0	−2.95	CZ	8.0	7.6	−5.1	−0.33	LU	1.89	2.89	53.1
Czechia (CZ)	23.5	17.9	−23.7	−1.68	AT	8.3	4.7	−43.3	−3.48	LV	2.38	2.02	−14.9
Lithuania (LT)	24.2	36.6	51.1	2.61	DE	9.0	5.5	−38.6	−3.00	MT	7.00	3.75	−46.5
Bulgaria (BG)	24.3	29.7	22.3	1.26	LT	10.3	14.6	41.7	2.20	NL	2.00	2.03	1.6
Austria (AT)	25.2	13.8	−45.3	−3.70	HR	10.3	4.4	−56.9	−5.13	PL	3.52	2.84	−19.4
Estonia (EE)	28.6	33.2	16.2	0.94	SK	10.9	12.1	10.8	0.64	PT	3.10	4.11	32.4
Croatia (HR)	37.2	20.9	−43.7	−3.53	LU	11.6	4.8	−58.6	−5.37	RO	2.37	2.47	4.5
Slovakia (SK)	43.3	34.3	−20.8	−1.45	EE	11.8	11.4	−3.1	−0.19	SE	1.79	1.97	10.3
Slovenia (SI)	43.4 *	15.3	−64.7	−6.30	SI	17.1	3.2	−81.1	−9.87	SI	2.54	4.73	86.4
Romania (RO)	52.8 *	41.8	−20.8	−1.45	RO	22.3 *	16.9 *	−24.1	−1.72	SK	3.97	2.84	−28.5
Hungary (HU)	100.3 *	34.4	−65.7	−6.47	HU	30.6 *	9.9	−67.5	−6.79	UK	1.97	1.90	−3.4
Coefficient of variation (CV) %	92.0	80.2			CV, %	81.9	82.8						

Source: Own calculations based on Eurostat database [20]. The SDR and the gender disparity ratio ordered from min to max values in 2000. Outlier values marked by asterisks (*). ^1^ Gender disparity ratio: (SDR_Male_)/(SDR_Female_).

**Table 6:** Premature SDR for diabetes mellitus in the EU countries in 2000 and 2016 ^1^.

EU	SDR−Males in		Relative Change in %		EU	SDR-Females in		Relative Change in %		EU	Gender Disp. Ratio		Cumulative
States	2000	2016	Cumulative	Annualized	States	2000	2016	Cumulative	Annualized	States	2000	2016	Change, %
Greece (EL)	1.9	4.0	108.9	4.71	EL	1.5	2.2	45.3	2.36	LU	0.67	3.44	415.3
Luxembourg (LU)	2.2	2.9	32.7	1.79	IE	1.6	0.9	−42.5	−3.40	MT	0.81	0.92	13.7
Malta (MT)	2.9	5.3	83.1	3.85	ES	1.8	0.8	−53.3	−4.65	LV	1.05	1.67	59.4
United Kingdom (UK)	2.9	2.7	−8.6	−0.56	AT	1.9	2.1	12.1	0.72	LT	1.07	2.58	140.7
Spain (ES)	3.6	2.3	−37.2	−2.87	FI	1.9	1.5	−20.5	−1.43	RO	1.08	1.89	75.2
Austria (AT)	3.6	5.3	48.1	2.48	UK	1.9	1.6	−17.9	−1.22	EE	1.08	3.33	207.3
Ireland (IE)	3.8	1.9	−49.5	−4.18	BE	2.0	0.8	−58.3	−5.31	BG	1.17	1.70	44.8
Estonia (EE)	3.9	6.0	52.8	2.69	DE	2.1	1.8	−15.2	−1.03	EL	1.27	1.82	43.8
Belgium (BE)	4.0	2.8	−31.4	−2.33	FR	2.2	1.4	−34.3	−2.59	PT	1.34	1.77	32.0
Romania (RO)	4.1	4.6	12.9	0.76	CZ	2.4	2.7	12.1	0.72	HU	1.49	2.10	40.8
Sweden (SE)	4.1	4.1	−0.2	−0.02	SE	2.4	1.7	−27.9	−2.03	UK	1.53	1.70	11.3
Finland (FI)	4.3	3.6	−17.2	−1.17	EU-28	2.6	1.8	−30.3	−2.23	PL	1.63	2.50	53.0
Lithuania (LT)	4.4	4.3	−1.4	−0.09	IT	2.8	1.7	−40.0	−3.14	SE	1.71	2.36	38.4
Latvia (LV)	4.5	7.2	60.9	3.02	HR	2.9	3.2	9.6	0.57	SK	1.74	2.12	21.7
Czechia (CZ)	4.6	5.9	28.5	1.58	SI	3.1	1.3	−57.7	−5.24	NL	1.85	2.46	33.0
France (FR)	4.6	3.3	−27.9	−2.03	LU	3.3	0.9	−74.2	−8.13	IT	1.86	2.37	27.6
Germany (DE)	4.9	4.5	−8.4	−0.54	NL	3.3	1.4	−56.4	−5.05	AT	1.89	2.50	32.1
EU-28	5.0	4.1	−18.5	−1.27	EE	3.6	1.8	−50.3	−4.27	EU-28	1.90	2.23	17.0
Italy (IT)	5.2	4.0	−23.5	−1.66	MT	3.6	5.8 *	60.8	3.03	CZ	1.92	2.20	14.6
Portugal (PT)	5.5	4.4	−20.2	−1.40	PL	3.8	2.7	−28.9	−2.11	CY	1.95	2.84	45.4
Croatia (HR)	5.9	6.4	7.5	0.45	RO	3.8	2.5	−35.5	−2.71	BE	1.99	3.27	64.3
Netherlands (NL)	6.1	3.5	−42.0	−3.34	SK	3.9	2.3	−40.8	−3.22	DK	2.00	2.27	13.4
Poland (PL)	6.2	6.7	8.7	0.52	LT	4.1	1.7	−59.0	−5.42	ES	2.00	2.69	34.5
Slovenia (SI)	6.7	2.2	−67.0	−6.70	PT	4.1	2.5	−39.5	−3.09	HR	2.03	1.99	−1.9
Slovakia (SK)	6.8	4.9	−27.9	−2.03	LV	4.3	4.3	0.9	0.06	FR	2.12	2.32	9.6
Hungary (HU)	7.9	8.3	5.2	0.32	CY	4.6	2.2	−52.2	−4.51	SI	2.16	1.69	−21.9
Bulgaria (BG)	8.1	5.8	−28.0	−2.03	DK	4.7	2.9	−38.1	−2.95	FI	2.26	2.36	4.2
Cyprus (CY)	8.9	6.2	−30.5	−2.25	HU	5.3	4.0	−25.3	−1.81	DE	2.33	2.52	8.1
Denmark (DK)	9.4	6.6	−29.8	−2.19	BG	6.9	3.4	−50.3	−4.27	IE	2.38	2.09	−12.1
Coefficient of variation (CV) %	37.5	39.8			CV, %	47.6	61.1						

Source: Own calculations based on Eurostat database [20]. The SDR and the gender disparity ratio ordered from min to max values in 2000. Outlier values marked by asterisks. ^1^ Gender disparity ratio: (SDR_Male_)/(SDR_Female_).

**Table 7:** Premature SDR for chronic lower respiratory diseases in the EU countries in 2000 and 2016 ^1^.

EU	SDR−Males in		Relative Change in %		EU	SDR-Females in		Relative Change in %		EU	Gender Disp. Ratio		Cumulative
States	2000	2016	Cumulative	Annualized	States	2000	2016	Cumulative	Annualized	States	2000	2016	Change, %
Cyprus (CY)	2.0	1.9	−3.0	−0.19	EL	1.0	1.0	−4.0	−0.25	DK	0.63	1.18	88.1
Greece (EL)	2.4	2.2	−9.2	−0.60	HR	1.1	2.4	125.4	5.21	SE	0.91	0.67	−26.9
Sweden (SE)	3.1	1.9	−38.1	−2.95	ES	1.3	1.3	3.1	0.19	CY	1.25	1.28	2.1
France (FR)	3.5	2.9	−15.8	−1.07	IT	1.3	1.0	−23.1	−1.63	NL	1.29	0.77	−39.9
Italy (IT)	3.5	2.1	−39.1	−3.06	FR	1.4	1.4	0.7	0.04	UK	1.34	1.15	−14.6
Portugal (PT)	5.1	3.8	−25.7	−1.84	EE	1.6	1.0	−37.5	−2.89	BE	1.85	1.36	−26.9
Finland (FI)	5.4	3.8	−29.8	−2.19	CY	1.6	1.5	−5.0	−0.32	IE	1.90	0.80	−57.8
Netherlands (NL)	5.8	4.3	−25.3	−1.81	LV	1.7	2.3	37.1	1.99	DE	2.23	1.52	−32.1
Croatia (HR)	5.9	5.6	−5.1	−0.32	SI	1.7	1.2	−31.8	−2.36	FI	2.25	1.91	−14.9
Spain (ES)	6.3	3.3	−47.8	−3.98	SK	1.7	2.8	65.3	3.19	EU-28	2.26	1.65	−27.1
EU-28	6.3	5.3	−16.1	−1.09	PT	1.9	1.2	−38.4	−2.98	EL	2.40	2.27	−5.4
Germany (DE)	6.7	6.7	0.3	0.02	BG	2.4	2.1	−12.1	−0.80	LU	2.46	0.69	−72.1
Austria (AT)	6.7	5.0	−24.9	−1.78	CZ	2.4	3.1	28.3	1.57	FR	2.47	2.06	−16.4
Denmark (DK)	7.7	7.9	2.5	0.15	FI	2.4	2.0	−17.5	−1.20	AT	2.58	1.65	−35.8
Czechia (CZ)	7.8	8.9	14.0	0.82	AT	2.6	3.0	16.9	0.98	HU	2.66	1.74	−34.7
Estonia (EE)	7.8	2.6	−66.4	−6.59	MT	2.7	1.0	−61.9	−5.85	PT	2.68	3.24	20.7
Ireland (IE)	8.0	3.0	−63.1	−6.04	EU-28	2.8	3.2	15.0	0.88	IT	2.69	2.13	−20.9
Latvia (LV)	8.0	7.6	−5.6	−0.36	DE	3.0	4.4	47.7	2.47	PL	2.84	1.94	−31.8
Slovakia (SK)	8.3	5.8	−29.8	−2.18	LT	3.0	1.1	−64.7	−6.30	CZ	3.25	2.89	−11.2
Bulgaria (BG)	8.7	5.1	−41.8	−3.33	RO	3.1	3.0	−3.5	−0.23	BG	3.63	2.40	−33.8
United Kingdom (UK)	9.0	7.7	−14.4	−0.97	PL	3.2	2.2	−32.5	−2.43	MT	3.63	1.99	−45.2
Luxembourg (LU)	9.1	3.0	−67.5	−6.78	SE	3.4	2.9	−15.3	−1.03	RO	4.13	4.03	−2.5
Poland (PL)	9.1	4.2	−54.0	−4.73	LU	3.7	4.3	16.8	0.97	LT	4.17	6.41	53.7
Slovenia (SI)	9.6	2.2	−77.2	−8.82	IE	4.2	3.7	−12.6	−0.84	LV	4.71	3.24	−31.1
Malta (MT)	9.8	2.1	−79.1	−9.32	NL	4.5	5.6	24.2	1.36	ES	4.85	2.46	−49.3
Belgium (BE)	9.8	6.6	−33.4	−2.50	BE	5.3	4.8	−8.9	−0.58	EE	4.88	2.62	−46.3
Lithuania (LT)	12.5	6.8	−45.7	−3.74	HU	6.4	11.7 *	82.8	3.84	SK	4.88	2.07	−57.5
Romania (RO)	12.8	12.0	−5.9	−0.38	UK	6.7 *	6.7	0.1	0.01	HR	5.55	2.34	−57.9
Hungary (HU)	17.0 *	20.3 *	19.2	1.11	DK	12.3 *	6.7	−45.5	−3.73	SI	5.65	1.89	−66.6
Coefficient of variation (CV) %	51.2	71.3			CV, %	82.3	73.0						

Source: Own calculations based on Eurostat database [20]. The SDR and the gender disparity ratio ordered from min to max values in 2000. Outlier values marked by asterisks (*). ^1^ Gender disparity ratio: (SDR_Male_)/(SDR_Female_).
